# Nanomaterial-related hemoglobin-based oxygen carriers, with emphasis on liposome and nano-capsules, for biomedical applications: current status and future perspectives

**DOI:** 10.1186/s12951-024-02606-1

**Published:** 2024-06-16

**Authors:** Kai Zhu, Lijun Wang, Yao Xiao, Xiaoyong Zhang, Guoxing You, Yuzhi Chen, Quan Wang, Lian Zhao, Hong Zhou, Gan Chen

**Affiliations:** 1 Academy of Military Medical Sciences, Beijing, 100850 China; 2 Department of Morphology Laboratory, Zhuhai Campus of Zunyi Medical University, Zhuhai, 519041 China

**Keywords:** Oxygen transport, Hemoglobin-based oxygen carriers (HBOCs), Nanomaterials, Hemorrhagic shock, Cancer

## Abstract

Oxygen is necessary for life and plays a key pivotal in maintaining normal physiological functions and treat of diseases. Hemoglobin-based oxygen carriers (HBOCs) have been studied and developed as a replacement for red blood cells (RBCs) in oxygen transport due to their similar oxygen-carrying capacities. However, applications of HBOCs are hindered by vasoactivity, oxidative toxicity, and a relatively short circulatory half-life. With advancements in nanotechnology, Hb encapsulation, absorption, bioconjugation, entrapment, and attachment to nanomaterials have been used to prepare nanomaterial-related HBOCs to address these challenges and pend their application in several biomedical and therapeutic contexts. This review focuses on the progress of this class of nanomaterial-related HBOCs in the fields of hemorrhagic shock, ischemic stroke, cancer, and wound healing, and speculates on future research directions. The advancements in nanomaterial-related HBOCs are expected to lead significant breakthroughs in blood substitutes, enabling their widespread use in the treatment of clinical diseases.

**Graphical Abstract:**

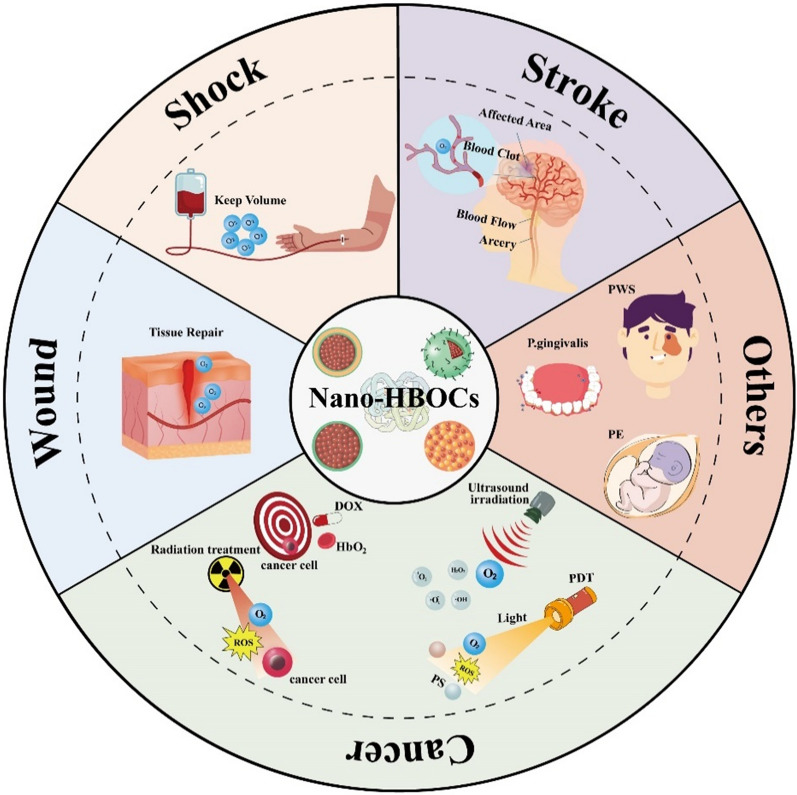

## Introduction

Oxygen (O_2_) is essential for life [1] and physiological processes such as cell growth, differentiation, metabolism, and tissue homeostasis [2, 3]. Factors such as trauma [4], blood loss [5], pernicious anemia [6] and ischemic cerebrovascular disease [7, 8] can decrease tissue oxygen content, lead to hypoxia [9]. This, in turn, can induce organ dysfunction, particularly in the brain [10], heart [11] and kidney [12] and leading to irreversible damage and/ or death [13]. Besides, the hypoxic microenvironment of tumor tissues can decrease the sensitivity of malignant tumors to antitumor drugs and enhance the invasion and metastasis abilities of tumor cells [14–16]. Thus, maintaining an adequate O_2_ supply to tissues and organs, is essential for maintaining the physiological functions of the body, and it is critical for disease treatment [17].

As natural oxygen carriers, red blood cells (RBCs) can bind to O_2_ in the lungs and deliver throughout to the body. This process is essential to meet the metabolic demands of the body [18, 19] and ensure normal organ function [20]. Hemoglobin (Hb), the main O_2_ carrier in RBCs, facilitates O_2_ delivery via its well-known oxygen-carrying function [21]. In adults, Hb consist of four subunits (α1, β1, α2, and β2) that form a tetrameric protein containing a central ferrous heme, enabling it to transport O_2_ by binding reversibly to O_2_ [22, 23].

Numerous factors, such as trauma, blood loss, and pernicious anemia [24, 25], can potentially cause decrease in RBCs and Hb levels in the bloodstream. Timely transfusion of RBCs, restoration of blood volume in the body, and ensuring adequate of O_2_ supply are essential for saving lives [26–28]. Indeed, these measures are widely practiced in clinical and military rescue scenarios [29]. However, the demand for natural blood often outpaces supply [30, 31]. Natural blood has a limited preservation period (< 42 d), requires special storage and transportation conditions [32], and is susceptible to damage and adverse reactions when infused [33, 34]. Natural blood transfusion also carriers risk of infectious diseases, requires associated, time-consuming, cross-matching [35, 36] and may conflict with religious beliefs [37, 38]. Therefore, blood substitutes that can fully and completely or partially replace the oxygen-carrying functions of RBCs have emerged. These substitutes can be classified into perfluorocarbons (PFCs) and hemoglobin-based oxygen carriers (HBOCs) [39, 40].

Perfluorocarbon emulsions, which physically solubilize O_2_ and CO_2_, were the first blood substitutes to be tested as oxygen carriers [41]. However, the biosafety and stability concerns of PFCs limit their potential for clinical use. The first-generation products, Fluosol-DA®, was originally approved by the United States of America Food and Drug Administration (FDA) for coronary transluminal angioplasty; however, it was withdrawn from the market due to O_2_ delivery capacity, poor stability, and complement activation [42]. The second-generation product, Oxygent™, was also discontinued during phase III clinical trials in the USA because of an increased incidence of instroke and heart diseases in patients undergoing coronary artery bypass surgery [42–44].

HBOCs are a class of blood substitutes that are based on natural hemoglobin obtained through polymerization, cross-linking, and modification with polymer. HBOCs have gradually become the primary research focus for artificial oxygen carriers because they closely resemble the natural oxygen-carrying/-releasing characteristics of Hb [40, 45]. HBOCs development progressed after decades of laboratory and clinical research. However, only a few HBOCs have received worldwide approved for clinical use. Glutaraldehyde-polymerized bovine hemoglobin (HBOC-201, BioPure company), was approved for the treatment of acute pernicious anemia in South Africa and Russia in 2001 and 2010, respectively [46, 47]. PEGylated carboxyhemoglobin bovine (Sanguinate, Prolong Pharmaceuticals, USA), was registered in 2015 under the FDA classification as an “orphan drug” for the treatment of sickle cell anemia; it is currently in phase III clinical trials [48]. Despite these advancement, HBOCs development is also complicated by issues such as vasoactivity caused by nitric oxide (NO) scavenging [49], nephrotoxicity from dissociated dimers [50], oxidative toxicity of hemoglobin [51], and short circulation time [52], resulting in many HBOCs varieties being discontinued or excluded from further research due to safety concerns. Thus far, researchers have focused on addressing the safety concerns of HBOCs by improving strategies such as the cross-linking technique, and the preparation process of Hb, as well as infusion methods. For instance, preventing the dissociation of hemoglobin tetramer and reducing nephrotoxicity [53, 54] by optimizing the molecular weight of the polymer [55]. Hb oxidation is reduced by co-cross-linking Hb with internal biological enzymes such as superoxide dismutase (SOD) and catalase (CAT) [56–58]. While these technical strategies have reduced the safety concerns of HBOCs to extent; they are still in the preclinical research stage.

The role of nanomaterials in drug delivery and disease treatment has received increased research attention due to the rapid development of nanotechnology [59, 60], which provides new opportunities for developing HBOCs. Nanomaterial-related HBOCs (Nano-HBOCs) are prepared by organically conjugating of nanomaterials with hemoglobin through encapsulation, self-assembly, bioconjugation, entrapment, and attachment [61–63]. This approach not only closely mimics the physiological structure of natural RBCs but also offers more advantages, such as reduced the vasoactivity, improved circulation time [64–66], and enhanced biological safety [67] (Table 1).

**Table 1:** Several advantages of Nano-HBOCs, classified into seven categories, with information related to their formulations, Hb source, and references

Properties	Categories	Formulations	Hb source	References
Reducing the vasoactivity	TRM-645	Encapsulating purified Hb in liposomes mimicking the RBCs membrane	Human	[69]
Raising biological safety	LEH HbPs HbTcMs O_2_@Hb@ZIF-8	Employment of liposomes to encapsulate intact Hb molecules Hb encapsulated mPEG-PLGA using the double emulsion technique Hemoglobin-polymer conjugate formation, completion of Hb and PS co-loading Hb encapsulated in ZIF-8	Human Bovine Bovine Human	[65, 70–72]
Improving blood circulation time	PDA − LtEc ZIF-8@Hb Hb@AuNCs PFRT-RBCS	*Lumbricus terrestris* with PDA surface-coating Hb encapsulated in MOFs Hb and Au nanoparticles, combined and incorporated into the reported MOFs A large amount of ZnF16Pc tethered to the RBCs surface	Worm Bovine Bovine Human	[73–76]
Enhancing antioxidant capacity	Hb-PDA PtNP MXene@PDA NSs	Combination of Hb and PDA using a template-based co-precipitation technique Platinum nanoparticles with SOD and CAT-mimetic enzyme activities combined with HSA on the outer layer Coupling of hyaluronic acid and PDA catalyzed by H_2_O_2_/HbO_2_ to encapsulated Ti_3_C_2_MXene nanosheets	Bovine Human	[77–79]
Mimicking physiology of RBCs	ErythroMer	Hb Encapsulated in deformable, hybrid peptidic-lipid nanoparticles	Human	[80]
Empowering O_2_ targetingly	CPTK@PMH HCM DHCNPNPs V(Hb)@DOX Au-Hb@ PLT NPs	Introducing PDA to noncovalently engage Hb and methoxatin to form PMH, and connecting to a specific fibrin-binding peptide Linking GLUT1, and bounding to the micelles via a condensation reaction to form Hb-conjugated micelles Hb and DOX encapsulated in PLGA Utilizing Hb via coupling ε-Caprolactone self assembles to form hollow V(Hb), and loading DOX into V(Hb) Coating Au-Hb NPs with PLT membrane	Human Bovine Human Bovine Human	[81–85]
Releasing O_2_ controllably	MN	Loading with black phosphorus and Hb	Bovine	[86]

**Fig. 1:**
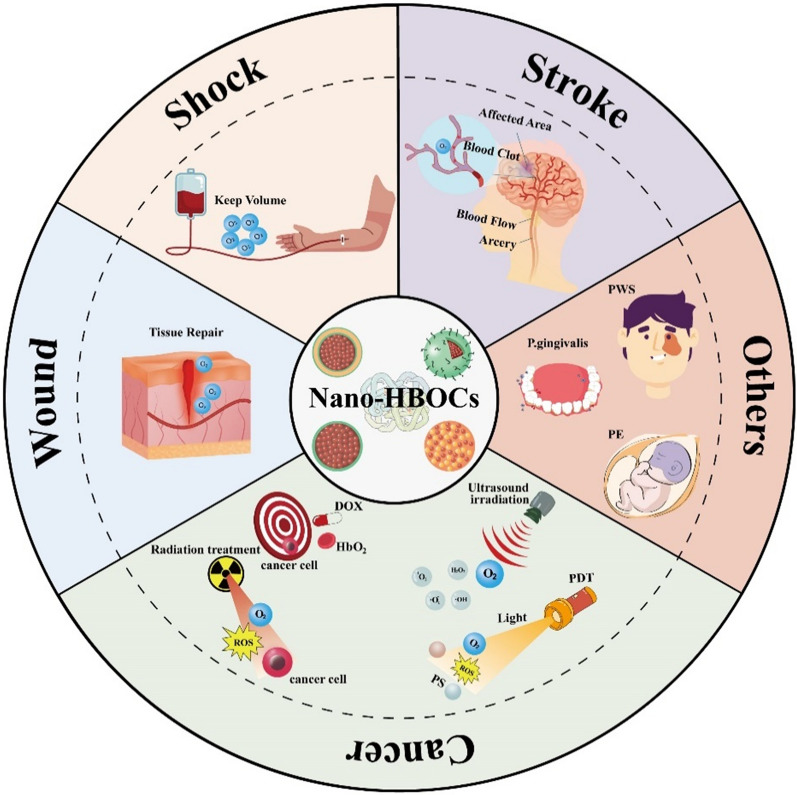
Schematic representation of the application of Nano-HBOCs in hemorrhagic shock, ischemic stroke, cancer, wound healing, and other disease treatment

Although some excellent reviews have discussed Nano-HBOCs [35, 45, 65–68], there is a lack of detailed and systematic introductions, which, coupled with the rapid progress in the research of Nano-HBOCs applications, has resulted in a lack of reviews on the latest advances in the preparation and application of Nano-HBOCs. In this review, we introduce the specific research on Nano-HBOCs in biomedical fields over decade. We focus on the applications of Nano-HBOCs in hemorrhagic shock, ischemic stroke, cancer therapy, and wound healing (Fig. 1). Additionally, we systematically summarize the research progress, current challenges and future prospects.

## Nano-HBOCs for hemorrhagic shock therapy

Hemorrhagic shock (HS) is a complex condition caused by a reduction in the effective circulating blood volume [87, 88], resulting in irreversible tissue hypoxia [89], eventually triggers circulatory failure [90]. Increasing the oxygen-carrying capacity to restore O_2_ supply to the tissue is a necessary approach to treating HS [88, 91] and perverting circulatory failure.

### Restoring oxygen supply

Liposome-encapsulated human hemoglobin (LEH) is a type of HBOCs prepared by encapsulating intact Hb molecules in liposomes, also known to as hemoglobin vesicles (HbV) [65]. LEH has been extensively researched as a potential treatment for HS in animal models [92–95]. Studies have shown that LEH has excellent biocompatibility [96], high biological safety [97–99], easy metabolism and excretion [100, 101], and low vasoactivity [102]. Researchers at the Terumo Research and Development Center [69] (Terumo Co., Tokyo, Japan) used a high-speed emulsification method to encapsulate purified human Hb in liposomes that mimic RBCs membranes. They modified the lipid membrane surface with PEG to form a nanoscale liposomal oxygen carrier (namely TRM-645). In a mouse hypohemoglobinemic shock model, the intravenous infusion of TRM-645 rapidly restored mean arterial pressure (MPA) from 30 to 70 mmHg; its effect on restoring and maintaining MAP was comparable to that of native RBCs. The intracavitary infusion of TRM-645 significantly improved overall survival [103] in a murine of HS induced by sustained massive hemorrhage, with a better treatment effect RBCs. TRM-645, due to its smaller size, is more likely to enter the circulatory system from the bone marrow cavity, rendering it an effective method for critical scenarios and pre-hospital emergency care. Vivek et al. [104] utilized non-phospholipid anionic lipid conjugated PEG containing hexadecyl carbamoyl methyl hexadecanoate (HDAS) to modify the surface of LEH. This modification improved the host’s tolerance to the immune response against LEH [105, 106], delayed its phagocytic clearance by monocytes, and increased the mean residence time in circulation. In a rat model of HS, LEH infusion restored blood volume, improved tissue oxygenation capacity, and ameliorated HS-induced systemic inflammation and multiple organ failure, suggesting that this modified LEH is a potential treatment for HS.

To address the critical need for a transportable, and temperature-stable blood substitute, a bio-synthetic, nano-artificial erythrocyte called Erythromer (EM) [107] has been developed. EM encapsulates human Hb within deformable, hybrid peptidic-lipid nanoparticles. This design, allows for a true physiological correlation between O_2_ affinity and tissue respiration. EM employs a novel shuttle, a small molecule allosteric effector, which adjusts P_50_ in response to pH changes, influencing Hb ~ O2 affinity. The use of EM allows for the mimicry of key physiological properties of natural RBCs, offering a promising solution to address the aforementioned critical need. Compared to the use of hydroxyethyl starch for resuscitation, EM maintains hemodynamics stability, increases arterial oxygen partial pressure, and improves acidosis in the HS rat and hemodilution mouse models [80]. Okamoto et al. [108] demonstrated that the combination of bovine hemoglobin (bHb) and human serum albumin (HSA) forms a core–shell protein called HbX-HSA_3_, which has high oxygen affinity. HbX-HSA_3_ facilitates O_2_ delivery to hypoxic tissues during HS. In the rat HS model, HbX-HSA_3_ effectively restores and maintains basic life indicators such as blood pressure, blood oxygen, respiration, and body temperature. It also reduces body lactate levels; and improve the survival rate of HS. Lu et al. [70] used methoxy polyethylene glycol-poly (D, L-lactide-co-glycolide) and mPEG-PLGA to encapsulate bHb using the double emulsion (w/o/w) technique for preparing hemoglobin-loaded nanoparticles (HbPs). This was based on the good biodegradability and biocompatibility characteristics of PLGA, which had a uniform particle size with stable oxygen-carrying function and excellent blood compatibility. In a controlled hemorrhage mouse model, HbPs maintained MAP, improved venous oxygen partial pressure, and restored tissue oxygen supply, demonstrating a promising application potential.

Polydopamine (PDA) has excellent biocompatibility and antioxidant capacity [109]. It can easily and efficiently adhere to the surface of various substrate materials [110], suggesting that PDA has the potential to serve as a universal modification platform for Hb. This can help address the issue of oxidative toxicity in HBOCs. Wang et al. [77] harnessed the unique characteristics of PDA to modify bHb and prepare Hb-PDA, which does not cause platelet aggregation or significant hemolysis. These novel Nano-HBOCs demonstrated outstanding oxygen affinity (P_50_ = 13.86 mmHg). Hu et al. [78] combined a template-based co-precipitation technique to develop Nano-oxygen carriers, (named Hb-PDA), which displayed uniform size and high biocompatibility. In vitro and in vivo experiments showed that Hb-PDA enhanced the antioxidant properties of Hb, alleviated the toxicity of free Hb, and maintained the O_2_ delivery capacity. Further, the resuscitation efficacy of Hb-PDA was investigated using in rat HS model. The results revealed that Hb-PDA maintained the stability of the blood pressure for a longer time relative to using saline alone. Baidukova et al. [111] modified bHb with PDA to synthesize morphologically homogeneous PD-HbMPs. These novel HBOCs showed antioxidant activity and enhanced Hb oxygen-binding capacity. Further, PD-HbMPs exhibited a high ability to scavenge free radical including H_2_O_2_, indicating their potential as practical HBOCs. Ethan et al. [73] proposed a photocatalytic method to synthesize the oxygen therapy agent PDA-LtEc using cell-free hemoglobin (Ec) from the PDA surface-coated worm *Lumbricus terrestris* (Lt). This method increased the oxidative protection of Ec during circulation, thereby making PDA-LtEc a promising oxygen therapy.

**Fig. 2:**
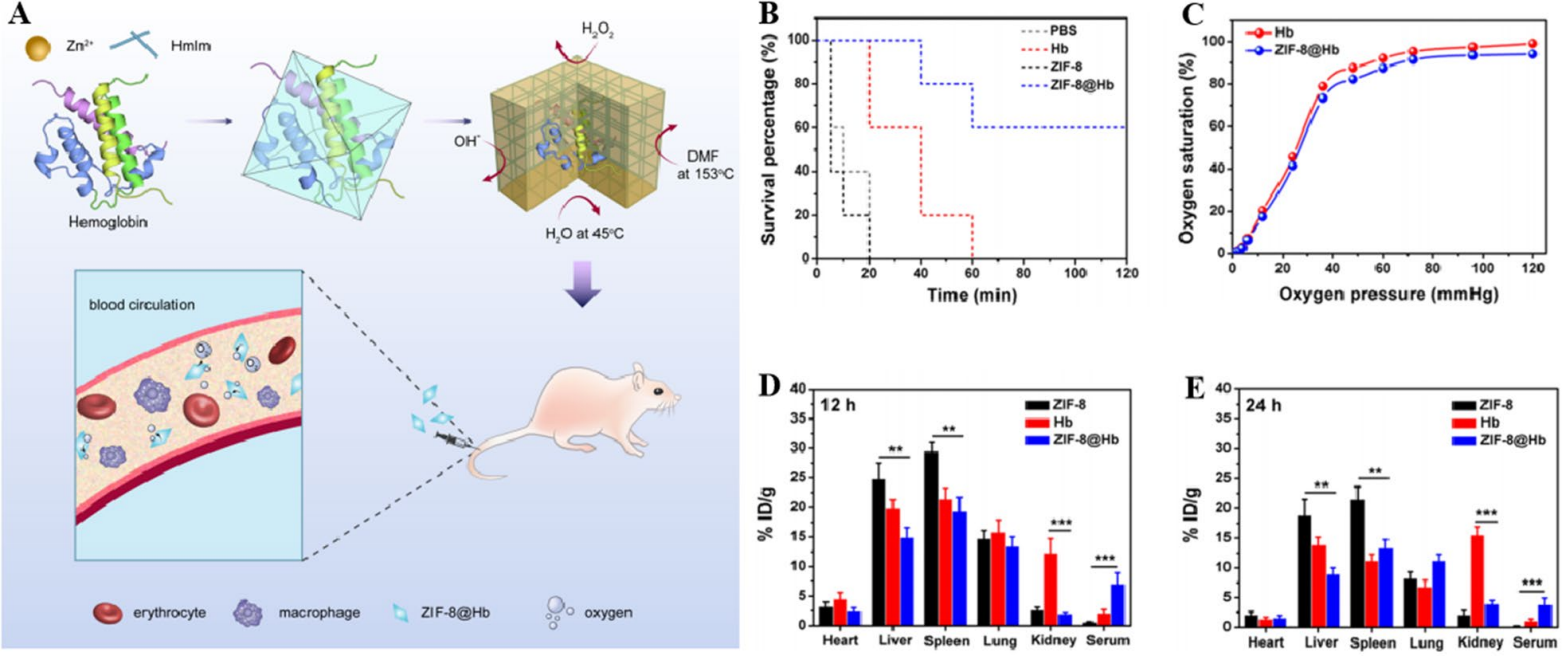
**A** Nanoparticle preparation, stability behavior, blood circulation, and oxygen supplying of ZIF-8@Hb are shown in figure. **B** Survival curves of mice with hemorrhagic shock following intravenous injection of PBS, Hb, ZIF-8, and ZIF-8@Hb. **C** Oxygen dissociation curves of Hb and ZIF-8@Hb. **D** Biodistribution of Hb, ZIF-8, and ZIF-8@Hb after intravenous injection for 12 h. **E** Biodistribution of Hb, ZIF-8, and ZIF-8@Hb after intravenous injection for 24 h. **A–E** Reproduced with permission [76]. Copyright 2019, American Chemical Society

Recently, metal–organic frameworks (MOFs; ZIF-8) have been widely used as protective coatings for functional biomacromolecules [112, 113] (such as DNA, proteins, and enzymes). This is because of their flexible topology and favorable physicochemical properties that improve stability during storage and manipulation [114–119]. Peng et al. [74] used MOFs to encapsulate bHb and created an oxygen-carrier platform with nanoporous coatings, ZIF-8@Hb. In vitro studies showed that the neutral charge state of the surface of ZIF-8@Hb surface significantly enhanced stability in alkaline, oxidative, elevated temperature, or enzymatic environments, which is favorable for long-term blood circulation (as the study found that the blood circulation half-life (t1/2) of ZIF-8@Hb was 13.9 h, compared to 1.4 and 5.1 h for ZIF-8 and Hb, respectively). Animal studies showed that intravenous injection of ZIF-8@Hb significantly prolonged the survival time of mice in an HS model. This study provides a highly stable and long-circulating oxygen-carrier platform for Nano-HBOCs (Fig. 2).

HS is an important application scenario for Nano-HBOCs. It is worth noting that HS treatment often requires large doses, and multiple intravenous infusion of Nano-HBOCs. Therefore, the safety requirements for Nano-HBOCs are extremely high. However, most current HS studies tend to focus only on the therapeutic effect and do not adequately evaluating biocompatibility, especially in the case of multiple and massive administrations. In this regard, a research group, from Denmark, developed the concept of “stealth properties” [120] of Nano-HBOCs, which may provide new ideas for the treatment of HS usig Nano-HOBCs.

## Nano-HBOCs for ischemic stroke therapy

Ischemic stroke is a clinical syndrome that refers to the disruption of blood supply in the brain caused by different cerebrovascular diseases, which eventually lead to local brain tissue ischemia, hypoxic necrosis, and corresponding neurological deficits [121]. Ischemic strokes are typically associated with varying degrees of cerebral infarcts and, in recent years, have become one of the leading causes of human mortality [122, 123] due to their increasing incidence and lethality. Ischemia and hypoxia in brain tissue cause impaired oxygen metabolism, which rapidly triggers brain tissue edema, focal neuronal cell function defects, and even necrosis [124, 125]. Following ischemia stroke, the blood flow in the tissue is typically restored within a short period, however, an excess of reactive oxygen species (ROS) and secondary inflammatory responses are generated in the body during ischemia stroke [126, 127]. These molecules cause tissue organ reperfusion injury [128], further exacerbating the ischemic stroke condition. Currently, several studies have indicated that early improvement in the hypoxic state of the infarct site can benefit ischemia stroke treatment [129, 130].

### Relieving cerebral infarction

HBOCs possess adjustable oxygen-carrying/-releasing capabilities and have a small particle size. These characteristics, which can enhance microvascular perfusion and collateral blood flow, lead to continuous improvement in oxygen supply to the ischemic area. As a result, they can effectively reduce cerebral infarct size [131, 132], providing a new choice for acute ischemic stroke treatment. Besides, Nano-HBOCs can inhibit glycolysis and lactic acid accumulation, which offers obvious advantages in reducing ischemic stroke injury and a significant protective effect on ischemic stroke tissue organs [133–136]. Komatsu et al. [137] established a rat middle cerebral artery occlusion (MCAO) model and an arachidonic acid (AA)-induced stroke models to evaluate the therapeutic effect of HbV. HbV treatment immediately after MCAO significantly reduced the infarct size (34.7%) in rats; the effect, was comparable to tthat of intravenous thrombolysis with a tissue plasminogen activator (tPA) in a thromboembolic model (34% reduction). The intravenous infusion of HbV reduced brain edema in an AA-induced thromboembolic stroke model. Kawaguchi [138, 139] et al. found that encapsulating Hb in liposomes prevented extravasation in rat stroke models; LEH significantly improved oxygen supply to the ischemic area and reduced cortical infarct size. Daiki Tomita [140] et al. covalently combined purified bHb and HSA to design a completely new oxygen-carrier platform with a core–shell structure to minimize the vascular leakage of HBOCs and increase circulation time in vivo (possibly through the effect of net surface charge)*.* This platform, known as HbX-HSA_m_, exhibits a high O_2_ affinity (P_50_ = 11.3 mmHg). Gekka [141] et al. covalently conjugated one Hb molecule with three human serum albumin (Hb1-HSA_3_) form HemoAct with a core–shell structure. In rat model of a transient MCAO (tMCAO), a significant increase in microvascular perfusion in the cortical penumbra and tissue oxygen partial pressure in the cortical penumbra were observed. Additionally, the treatment reduced liposome peroxidation, edema, and cerebral infarct size; while exerting potent neuroprotective effects in the treatment of transient ischemic injury encephalopathy via intravenous infusion. These studies suggest that improvement in the oxygen supply at the infarct site using infused Nano-HBOCs infusion effectively attenuates stroke injury.

**Fig. 3:**
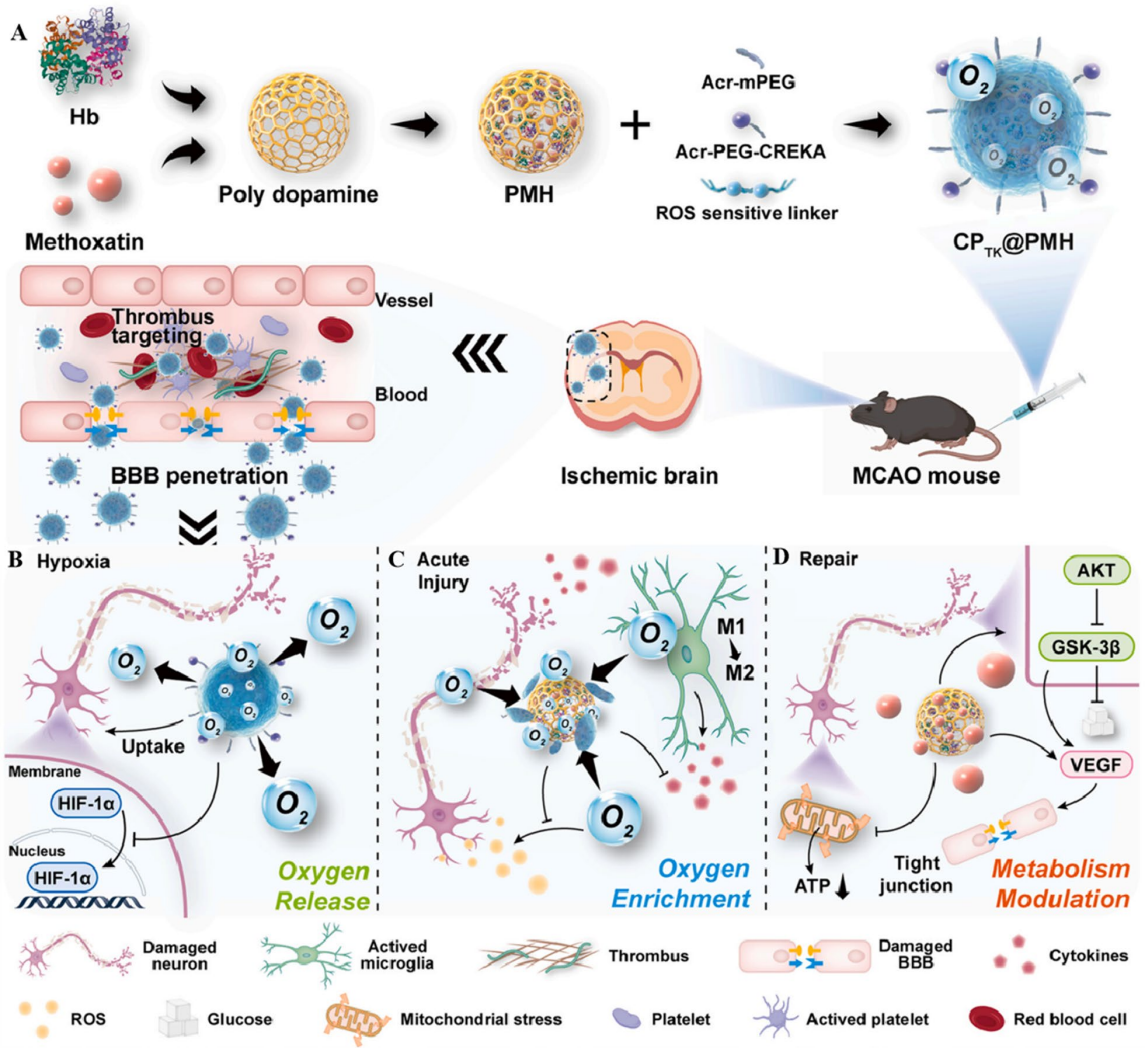
**A** Illustration of the CPTK@PMH nanoerythrocyte formation and metabolic microenvironment modulation in ischemic brain: nanoerythrocyte accumulation in ischemic core via microthrombus binding and uptake by neurovascular unit after blood brain barrier penetration; **B** hypoxia-responsive oxygen release to relieve necroptosis; **C** oxygen balance regulation to alleviate acute reperfusion injury via oxygen enrichment, ROS scavenging and microglia polarization; **D** repair promotion achieved by metabolic microenvironment modulation via energy and glucose metabolism activation and blood brain barrier protection. **A–D** Reproduced with permission [83]. Copyright 2019, Nano Today.

Nano-HBOCs can be used in multifunctional composites in combination with other functional materials boost their therapeutic efficacy. Hosaka [142] et al. synthesized covalent core–shell structural protein clusters called Hb-HSA based on purified bHb and HSA. Next, platinum nanoparticles with SOD and CAT-mimetic enzyme activities were combined with HSA on the outer layer of protein clusters to form Hb-HSA_3_ (PtNP) with antioxidant capacity. Based on the pathological microenvironment characteristic of stroke, Liu [81] et al. introduced PDA to noncovalently engage Hb and methoxatin (M) to form PMH, whose surface-coated ROS-sensitive linker (thioketal, termed TK linker) connects to a specific fibrin-binding peptide (CREKA, termed C-peptide), and they developed a bioinspired nanoerythrocyte (C-peptide-PRG-TK linker @PDA-Methoxatin Hb, referred to as CPTK@PMH). The half-life of free fluorescence labeled Hb was measured use a microplate reader, and the pharmacokinetic results showed that CPTK@PMH could significantly prolong Hb half-life from 12.045 ± 1.251 h to about 35 h. In a murine MCAO model, CPTK@PMH was shown to target the thrombus and ischemic site, release O_2_ in response to hypoxic signals, regulate the metabolic microenvironment, inhibit the oxidative stress injury, and reduce the cerebral infarct size during reperfusion, which ultimately protecting neurons from acute injury and improving neurological function after cerebral ischemia (Fig. 3).

### Inhibiting an inflammatory reaction

An inflammatory response is a key factor in the development and outcome of an ischemic stroke [143]. A series of inflammatory cascades are triggered after an ischemic stroke. Among these, microglial activation in brain tissue and neutrophil infiltration from peripheral blood into brain tissue are the hallmarks underlying microglial transformation from the M1 to M2 type. In particular, these processes inhibit neutrophil infiltration, which is a potential direction for treating cerebral ischemic injury [144]. Yin et al. [145] modified MG1 and RVG29 peptide chains based on erythrocyte-derived nanovesicles to prepare engineered nanoerythrocytes (NEMR) to promote microglial reprogramming. In an MCAO model, NEMR targeted the cerebral infarct site to attenuate tissue damage by delivering/-releasing oxygen, while inducing the overexpression of heme oxygenase-1 and CO production thriugh the Hb metabolism. This promotes microglial transformation into the M2 type, ultimately improving therapeutic outcomes in ischemic stroke. Furthermore, the modifications of MG1 and RVG29 peptides effectively prevented NEMR uptake immune cells in the bloodstream (including mononuclear macrophages), and improved the immune escape ability of NEMR. In addition, Shimbo et al. [146, 147] found that LEH inhibited neutrophil infiltration, matrix metalloproteinase-9 (MMP-9) [148] expression, and oxygen-free radical production, reduced cerebral infarct size and edema volume, and improved neurological indices in a rat MCAO model.

Although few studies have reported the applications of Nano-HBOCs in ischemic stroke, current research confirms that Nano-HBOCs can serve as an effective therapeutic option by targeting drug delivery and easily passing through the damaged blood–brain barrier, et al. However, stroke is a complex disease, and the unique advantages of Nano-HBOCs include their ability to relieve the ischemic and hypoxic state of brain tissues, co-load drugs, and ultimately achieve comprehensive treatment by combining thrombolysis, anti-inflammation and neuron protection. This approach also promotes rehabilitation, providing a diversified platform for treating of ischemic stroke.

## Nano-HBOCs for cancer therapy

Hypoxia is a common feature of most malignant tumors. Cancer cells proliferate in a disorderly manner and consume large amounts of oxygen [149, 150], which results in a tumor microenvironment (TME) [151–153] that is characterized by hypoxia accompanied by weak acids and high levels of H_2_O_2_, and it directly contributes to cancer cell metastasis, invasion, and angiogenesis [154, 155]. Hypoxic TME decreases the antitumor efficacy of chemotherapy, radiation therapy (RT), photodynamic therapy (PDT), and sonodynamic therapy (SDT) [156–160]. As oxygen carriers, Nano-HBOCs showed excellent stability and oxygen supply capacity, improved O_2_ delivery efficiency in hypoxic regions [161], alleviated the hypoxic state of tumors, and enhanced antitumor efficacy.

### Increasing drug sensitivity

The increased expression of hypoxia-inducible factor-1α (HIF-1α) in TME facilitates the adaptation of tumor cells to hypoxia [162, 163]. This is attributed to the development of chemical resistance that promotes the expression of p-glycoprotein (P-gp), which leads to chemotherapy failure [164, 165]. Therefore, improving the hypoxic environment of tumors is important to enhance the efficacy of chemotherapy. Kawaguchi et al. [166] reported that liposomes encapsulating hemoglobin (h-LEH) with high O_2_ affinity improved the oxygenation capacity of tumor cells in a mouse rectal cancer (Colon26) model. h-LEH combined with chemotherapeutic drugs by inhibiting HIF-1α activity, which prevented angiogenesis and inhibited tumor growth and metastasis. Human organic cation transporter 2 (OCT2) abnormalities confer renal cell carcinoma (RCC) increased resistance to chemotherapeutic agents such as oxaliplatin (OXA) and decitabine (DAC) under hypoxic conditions [167, 168]. To tackle this issue, Chen [169] et al. developed a simple and efficient oxygen nanocarrier based on Hb (H-NPs) combined with DAC. The H-NPs increased the oxygen content of the RCC tissue, alleviated hypoxia-induced loss of DAC activity, and enhanced the sensitivity of RCC cells to the combined treatment with DAC and OXA.

#### Targeted drug releasing

**Fig. 4:**
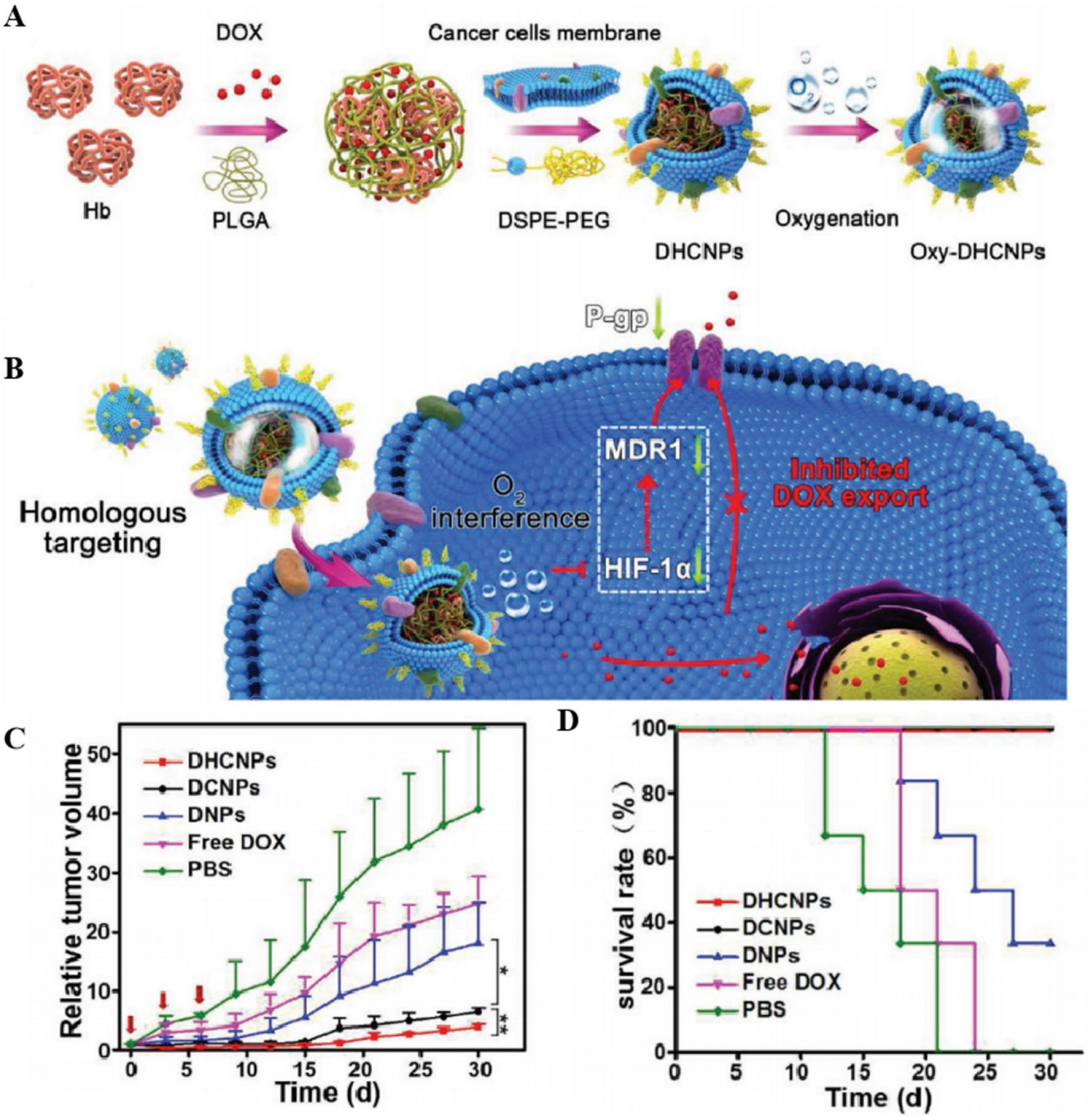
The design and function of DOX/Hb loaded PLGA-cancer cell membrane nanoparticles (DHCNPs) for homologous targeting and O2 interference. **A** Synthesis of oxy-DHCNPs. DHCNPs were prepared by extrusion with preformed DOX/Hb-PLGA NPs, DSPE-PEG, and MCF-7 cancer cell membrane, and then were oxygenated to obtain oxy-DHCNPs. **B** Cellular functions of DHCNPs, including homologous targeting, downregulation of predictive markers (HIF-1α, MDR1, and P-gp), and inhibited DOX export. (**C**): MCF-7 tumor growth curves of different treated groups (scale bar 50 µm). (**D**): Survival rates of tumor-bearing mice in various groups. **A–D** Reproduced with permission [85]. Copyright 2017, Advanced Functional Materials.

A hypoxic environment causes the overexpression of glucose transporter isoform 1 (GLUT1) [170] on the surface of cancer cells, and nanocarriers modified with glucose can identify cancer cells via GLUT1. Thus, Bu et al. [171] designed Nano-HBOC with both oxygen-carrying capacity and cancer cell-specific recognition. The synthesis of the triblock copolymer poly [2-(methacrylamido) glucopyranose]-b-poly (methacrylic acid) -b-poly (butyl methacrylate) (PMAG-b-PMAA-b-PBMA) micelles indicated that Hb was bound to the micelles via a condensation reaction to form Hb-conjugated micelles (HCM). PMAG-linked GLUT1 specifically recognizes cancer cells, and HCM can bind O_2_. Meanwhile, the uptake of DOX-loaded HCM by tumor cells was increased in a model of human cervical cancer cells (HeLa), and Nano-HBOCs were confirmed to target cancer cell recognition and reduce tumor drug resistance, ultimately improving therapeutic efficacy. Tian et al. [83] designed and constructed camouflaged biomimetic nanocomposites (DHCNPNPs) based on PLGA encapsulated with Hb and DOX. They modified the surface of the cancer cell membrane to realize homologous recognition. The DHCNPNPs exhibited strong self-recognition ability, affinity for homologous cancer cells in a model of human breast cancer cells (MCF-7 cells), and minimal toxicity to normal tissues. DHCNPNPs achieved O_2_-independent conditions and high target selectivity in the TME by inhibiting HIF-1α, down-regulating the expression of multidrug resistance gene 1 (MDR1) and P-gp, and increasing chemosensitivity, which improved the overall therapeutic efficacy (Fig. 4). Yang et al. [172] designed a multifunctional liposome, DOX-Hb-lipo (DHL), which was simultaneously loaded with human hemoglobin (hHb) and DOX. They showed that DHL could precisely release DOX and deliver O_2_ to specific hypoxic sites, thereby alleviating hypoxia-induced chemoresistance. DHL inhibited tumor growth in mouse breast cancer models (4T1) and mouse colon cancer models (CT-26), achieving in situ O_2_ release and relieving the hypoxic state of the TME, thereby providing an effective alternative for cancer treatment. Wang et al. [84] engineered an endogenous Nano-HBOCs called V(Hb)@DOX, which self-assembled resulting DOX-loaded biomimetic into hollow nanovesicles. In the acidic intertumoral environment, V(Hb)@DOX targeted M2-type macrophages and released DOX, thereby effectively suppressing of tumor growth and metastasis. V(Hb)@DOX prolonged the survival of mice bearing 4T1 orthotopic breast tumors and achieved long-lasting specific immunity in a murine colon cancer CT26 model.

### Promoting ROS generation

ROS are derived from O_2_ and are formed through oxidative reactions or electron excitation, they mainly consist of hydroxyl radicals (•OH), superoxide anion (•O-2) and singlet oxygen (^1^O_2_) [173]. When the levels of ROS exceed a certain threshold, they can disrupt the cellular structure and thus be used to inhibit tumor growth [174]. Nano-HBOCs can promote ROS production in three ways. Firstly, Nano-HBOCs can provides sufficient O_2_ as a feedstock for ROS production [175], while improving the TME. Furthermore, in the presence of ferrous ions (derived from Hb), high levels of H_2_O_2_ in the TME undergo a Fenton and Haber–Weiss reaction, which continuously produce highly toxic •OH [176]. Additionally, Hb can mimic horseradish peroxidase (HRP), which also produce •OH from H_2_O_2_ [177].

#### Application of Nano-HBOCs in RT

**Fig. 5:**
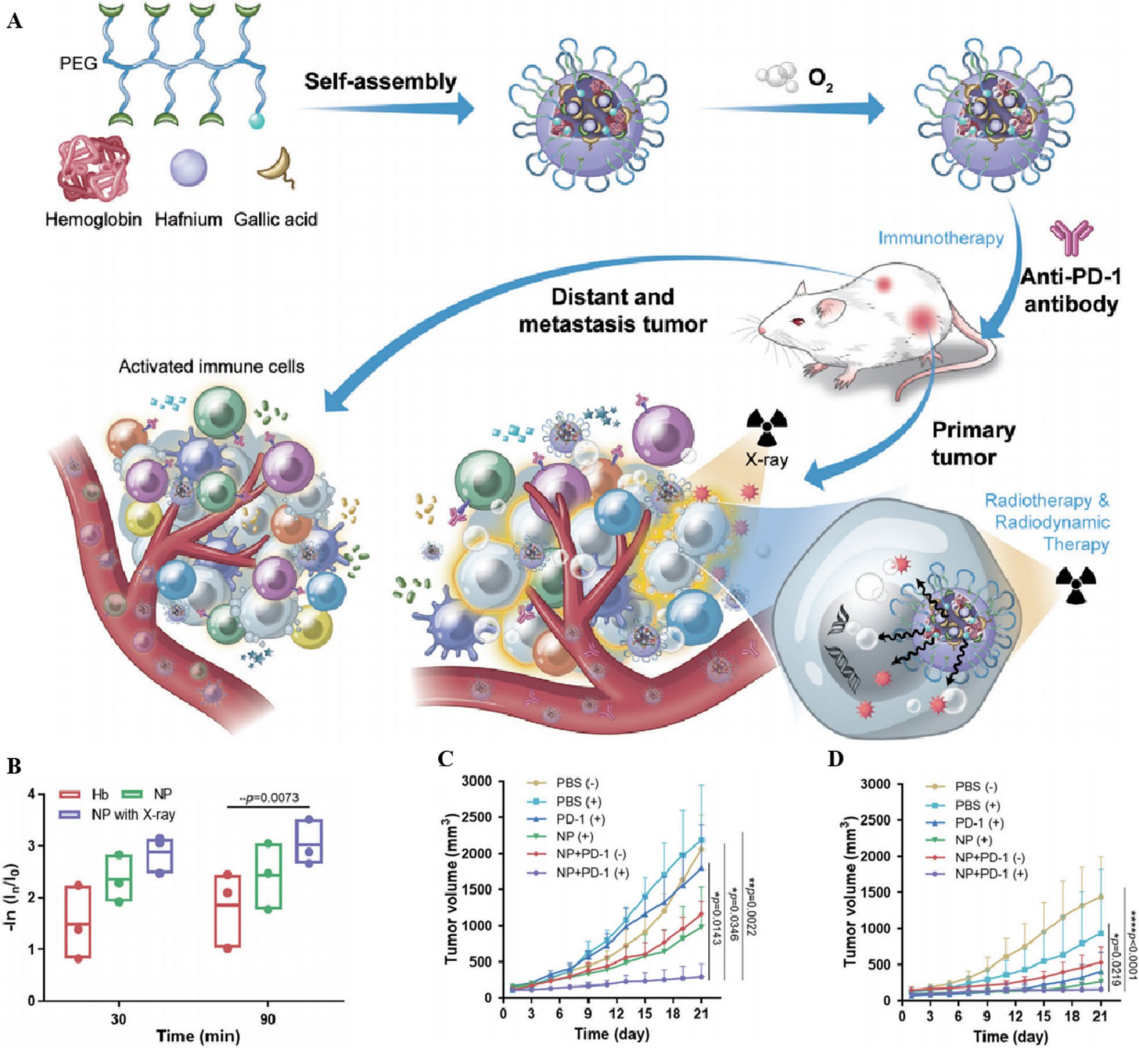
**A** Schematic illustration showed the Hb@Hf-Ce6 nanoparticles-mediated X-ray induced radiotherapy-radiodynamic therapy-immunotherapy for the eradication of both primary and distant tumors. Hb was encapsulated in the Hf-phenolic coordination nanoplatform for oxygen delivery through self-assembly. **B** The oxygen release behavior of hemoglobin (Hb) and Hb@Hf-Ce6 NPs (NP) with/without X-ray irradiation was evaluated by Ru(bpy)_3_Cl_2_ probe. Hb and NP were oxygenated previously. **C** Tumor volume growth curves for primary tumors. **D** Tumor volume growth curves for distant tumors. **A–D** Reproduced with permission [184]. Copyright 2021, Advanced Science.

RT, a common method for cancer treatment, damages DNA using X-rays and generates ROS to inhibit tumor growth; its therapeutic effect is directly related to the O_2_ content in the TME [178, 179]. Sang et al. [180] developed an oxygen-enriched X-ray nanoprocessor (Hb@Hf-Ce6 NPs) based on metal-phenolic coordination to modulate the oxygen balance in the TME and revere immunosuppression for cancer eradication and metastasis inhibition. In the nanoprocessor, the radiosensitizer hafnium (Hf) coordinated with chlorin e6 (Ce6) nanocomplexes and encapsulated Hb to deliver O_2_. Under X-ray irradiation, Hf emitted radioluminescence to activate Ce6 for ROS generation, and Hb@Hf-Ce6 NPs released O_2_ at the tumor site. This promoted T cell-mediated systemic immunity to exert long-range antitumor effects on tumors and enhance RT efficacy, thereby exhibiting superior antitumor efficacy in a bilateral tumor model and a lung metastatic model of 4T1 breast cancer. (Fig. 5). Xia et al. [85] combined a Hb oxygen carrier with gold nanoparticles (Au) radiosensitizer to prepare Au-Hb NPs. Inspired by the fact that P-selectin on the surface of platelets (PLT) can specifically bind to CD44 on the surface of cancer cells [181], platelet-coated oxygen-carrying nanoparticles (Au-Hb@ PLT NPs) were synthesized by coating Au-Hb NPs with PLT membrane. Au-Hb@PLT NPs could deliver sufficient O_2_ deep into the tumor tissue, relieve hypoxia, and improve the effect of RT. Au acts as a sensitizer, which enhances the sensitivity of tumor cells to X-rays and their therapeutic effects, showing good tumor-therapeutic efficiency and a few side effects in the HeLa cell model. Of note, a clear boundary between the normal parenchyma and dead cancer cells was confirmed using the hematoxylin and eosin staining (H&E), suggesting that Au-Hb@PLT + RT (2 Gy) killed tumor cells while causing relatively less damage to healthy tissue.

#### Application of Nano-HBOCs in PDT

Photodynamic therapy (PDT), a clinically recognized and minimally invasive tumor treatment, has become an alternative to chemotherapy [182, 183]. PDT includes a photosensitizer (PS), a light source, and sufficient O_2_. Under PS catalysis, O_2_ is activated to ROS, such as •OH and ^1^O_2_, which in turn induce tumor vascular injury, thereby leading to tumor cell death [184–186]. However, the hypoxic state of the TME impairs PDT efficacy [187], which can be effectively reversed by increasing the O_2_ content of the tumor [188, 189]. Wang et al. [190] developed a new PDT therapy based on the concept of O_2_ “self-compensation ability,” which was synthesized by chemically conjugating Hb with polymeric micelles formed by triblock copolymers of poly (ethylene glycol)-block-poly (acrylic acid)-block-polystyrene (PEG-b-PAA-b-PS). They used Hb as a PS zinc phthalocyanine (ZnPc) carrier, which endowed the Hb-conjugated ZnPc PS carrier with good oxygen-binding capacity, antioxidant activity, and the ability to produce abundant ^1^O_2_, thereby exhibiting robust photocytotoxicity in an in vitro HeLa cell assay.

**Fig. 6:**
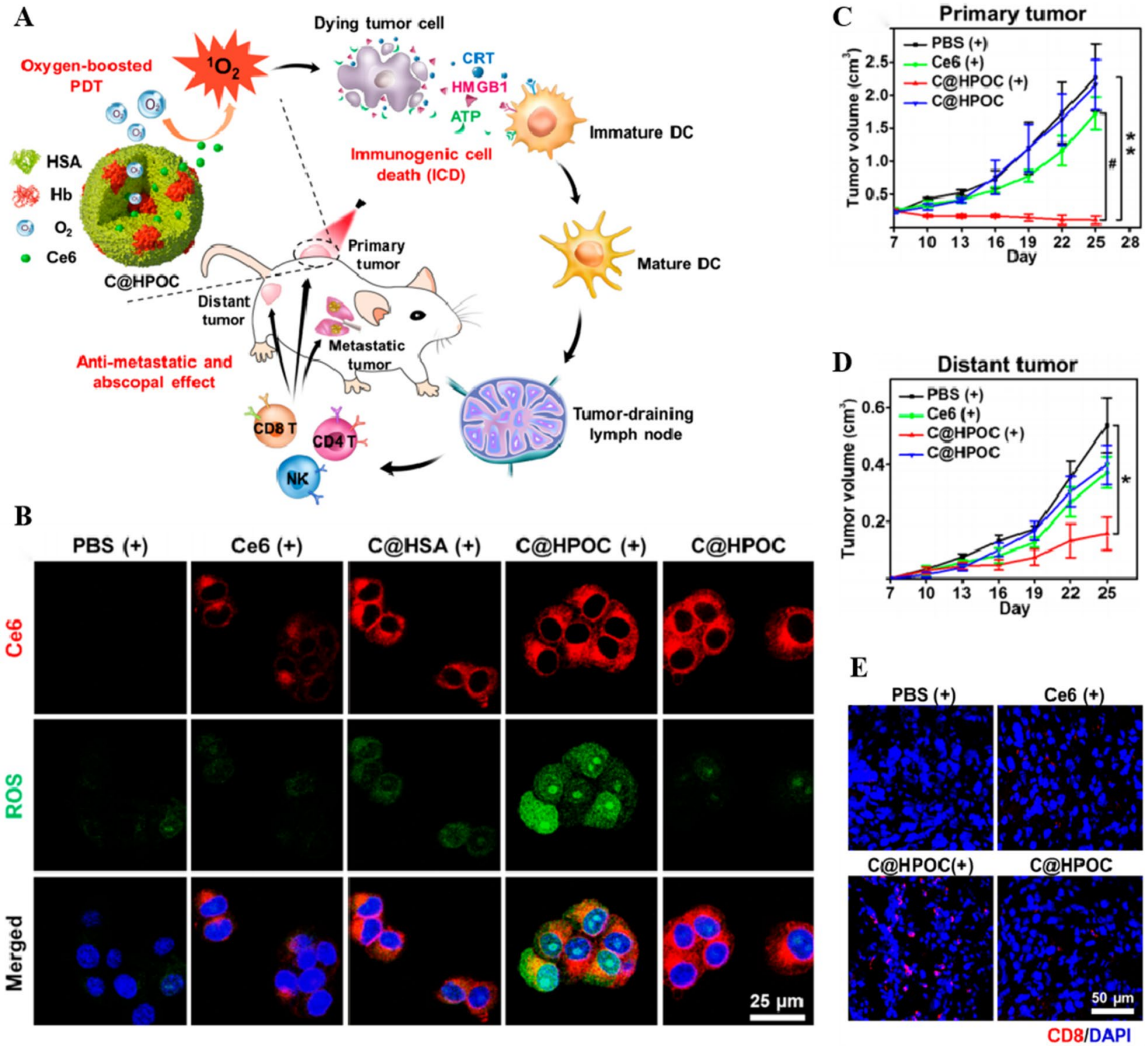
**A** Schematic depiction of oxygen-augmented immunogenic PDT with C@HPOC for eliciting the anti-metastatic and abscopal effect. Human serum albumin (HSA) was hybridized with oxygen carrying hemoglobin (Hb) via intermolecular disulfide bonds to form a hybrid protein oxygen nanocarrier with Ce6 loaded (C@HPOC). Under laser irradiation, oxygen self-supplied nanoparticles (C@HPOC) elevated the generation of cytotoxic ^1^O_2_ and moreover triggered immunogenic cell death (ICD). C@HPOC-mediated PDT not only destroyed the primary tumors but also inhibited the distant tumors and lung metastasis by systemic anti-tumor immune responses. **B** Confocal images of cellular uptake and ROS generation in 4T1 tumor cells.** C** Growth curves of primary tumor on mice after various treatments. **D** Growth curves of distant tumor on mice in different treated groups. **E** Immunofluorescence staining detection of CD8 T cells (red) in tumor tissues. **A–E** Reproduced with permission [197]. Copyright 2018, American Chemical Society Nanomaterials.

To address the low O_2_ levels in tumor and enhance PDT efficacy, Tang et al. [76] used ferritin, a protein-based nanocage, to tether a large amount of PS (such as ZnF16Pc) to the surface of RBCs. They utilized Hb as a PS carrier to provide O_2_, loaded ZnF16Pc at ferritin molar ratio (1:40) to form P-FRT, and simultaneously produced PFRT-RBCS nanoparticles by coupling RBCs. PFRT-RBCS prolonged the circulation half-life of Ps, which were labelled with IRDye800 that exhibited a high fluorescence in the blood (7.6 ± 2.1%ID g^−1^ at 1 h), and the signal remained strong for 24 h (3.2 ± 0.4%ID g^−1^ at 24 h). P-FRT-RBCs achieved continuous oxygen supply under hypoxia in the TME, thereby enhancing the ability of PDT to kill cancer cells in a U87MG tumor-bearing mouse model (human brain astroblastoma cells). Luo et al. [191] developed a biomimetic lipid-polymer nanoparticle loaded with PS (indocyanine green, ICG) and oxygen carrier (Hb). Nanoscale artificial RBCs (I-ARCs) were prepared by encapsulating Hb/ICG complexes in a PEG-modified lipid layer. In an MCF-7 human breast cancer model, the I-ARC provided sufficient O_2_, and ICG in the I-ARC efficiently converted O_2_ to ROS under near-infrared (NIR) laser irradiation, thereby triggering sustained damage to tumor cells. Furthermore, I-ARC serves as an ideal fluorescence imaging probe to dynamically monitor the biodistribution of nanoparticles and oxygen levels. The dynamic PDT process can be self-monitored through ICG, while serving as a diagnostic platform for self-monitoring therapy integration. Xu et al. [71] prepared a novel hemoglobin-polymer conjugate (HbTcMs) with high biocompatibility and stability as a carrier for both O_2_ and PS to provide additional O_2_ to enhance PDT efficacy. Compared with free Hb, HbTcMs not only retained the Hb oxygen-binding capacity but also exhibited higher stability against oxidation and trypsin digestion. In a 4T1 cell model, HbTcMs produced numerous oxygen-derived ^1^O_2_ molecules and exerted enhanced phototoxicity by utilizing the oxygen supply ability of Hb. Cao et al. [192] constructed the BP@RB-Hb nanoparticles by assembling Hb into nanocomplexes with novel two-photon species: bis(pyrene) (BP) and traditional PS (RB). Hb acts as an O_2_ donor to provide additional O_2_ resistance to the TME at the tumor site through NP targeting. Simultaneously, the resonance energy transfer effect (FRET) inside the nanoparticles indirectly stimulated PS under irradiation with a two-photon laser, thereby improving the treatment depth. In a tumor-bearing mouse model, the material showed excellent oxygen-carrying performance and penetration depth, greatly enhancing PDT efficiency. In tumor-bearing mouse models, BP@RB-Hb nanoparticles exhibited excellent penetration depth and oxygen-carrying properties, which greatly improved PDT treatment efficiency. Chen et al. [193] proposed a protein hybridization method for combining HSA and Hb to develop a bioinspired hybrid protein oxygen nanocarrier (C@HPOC) loaded with photosensitizer e6 (Ce6), which overcomes tumor hypoxia via co-delivery of tumor-targeted O_2_ and PS and improves PDT efficacy by generating a large amount of ^1^O_2_. C@HPOC stimulated robust systemic antitumor immunity and elicited potent antitumor metastatic and abscopal effects in a murine triple-negative breast cancer (4T1-mTNBC) model [194] (Fig. 6). Tian et al. [195] connected Hb to Ce6 to load sorafenib (SRF, an iron death promoter) and built a nanoplatform SRF@Hb-Ce6. Hb not only provides O_2_ and increases PDT efficacy, but it also synergizes with SPF, significantly increases the generation of lipid peroxides, downregulates GPX4 expression, and increases tumor cell iron death [196]. This platform shows promise as a nanoplatform for combination cancer therapy.

#### Application of Nano-HBOCs in SDT

SDT is an emerging cancer therapy that triggers ROS generation from abundant ^1^O_2_ using ultrasound (US), which leads to oxidative damage in tumor cells and enhanced therapeutic efficacy [197]. Its advantages include superior tissue penetration ability, minimal damage to surrounding healthy tissues, and lower skin sensitivity. The process of ROS generation using US irradiation of sonosensitizers during SDT requires the participation of O_2_, which is similarly limited by hypoxia in the TME [198]. In this context, Yin et al. [199] designed a novel intensified oxygen supply sonosensitizer system (MnPcS@HPO) by reconstituting bHb and HSA through disulfide bonds and loading the sonosensitizer Mn-phthalocyanine (MnPcs). This system combines the oxygen-carrying capacity of Hb and the tumor-targeting properties of HSA. MnPcS@HPO was enriched in the hypoxic area of the tumor, effectively alleviating the hypoxic state of the tumor. Simultaneously, MnPcS produces a large amount of ^1^O_2_ under US irradiation, effectively inhibiting tumor growth. Yuan et al. [72] constructed O_2_@Hb@ZIF-8 (OHZ) nanoparticles formed from ZIF-8 encapsulated Hb, which not only provided abundant O_2_ for US-induced ROS generation, but also showed excellent biocompatibility, as evidenced by cell viability evaluation and imagine (H&E) of major organs. ZIF-8 was used as a drug carrier for improving the Hb packaging rate, achieving pH-responsive Hb/O_2_ release, generating a large amount of O_2_ in the acidic TME, and relieving tumor hypoxia, thereby providing a source of O_2_ for US-triggered ROS generation. In 4T1 tumor-bearing mice, OHZ nanoparticles could not only inhibit the growth of subcutaneous tumors but also have an excellent inhibitory effect on the growth of deep-seated tumors, which helped achieve the SDT treatment of tumors at different depths. Pan et al. [200] synthesized ZIF-90 in water under mild conditions to encapsulate bHb and prepare an oxygen carrier, ZIF-90@Hb. The dynamics of oxygen consumption showed that the initial current of ZIF-90@Hb fanged from 9.15 μA to 4.18 μA, while that of free Hb was 2.78 μA and dropped to 0.55 μA within 100 s. This observation indicated that ZIF-90@Hb demonstrated better oxygen carrying/releasing abilities than free Hb. Furthermore, Cun et al. [75] similarly combined bHb and Au nanoparticles to synthesize ultrasmall protein metal clusters (MNCs) to develop a novel HBOC. They combined the complexes of Hb@AuNCs incorporated into the reported MOFs, which increased their antioxidant capacity to inhibit the formation of methemoglobin (the fluorescence intensity of hydrogen peroxide decreased sharply by 70%, with a remarkable 39% decrease in methemoglobin). Overall, these studies suggest that the utilization of the ZIF metal framework in oxygen carriers [74] provides a multifunctional platform for the development of blood substitutes in the near future. Liang et al. [201] encapsulated PtIV, a platinum (Pt) prodrug, with Hb to prepare a multimodal nanoparticle for tumor-targeted US radiation-triggered cancer therapy, harnessing the good solubility of Hb as a sonosensitizer. These nanoparticles can treat large volumes of deeply located tumors and provide new ideas for cancer treatment.

Hypoxia is a hallmark of TME in solid cancers [202]. It not only promotes tumor growth, but also reduces the effectiveness of treatment. Nano-HBOCs have been reported to have the ability to enhance synergistic therapy with stable O_2_ supply and can be loaded with drugs targeting tumor cells. Numerous studies have investigated the use of Nano-HBOCs in tumor therapy, and significant therapeutic effects have been observed in vitro and in vivo. By combining Nano-HBOCs with drugs and enhancing their abilities, we can potentially make a breakthrough in the clinical treatment of tumor.

## Nano-HBOCs for wound healing

O_2_ plays a key role in wound healing [203] by promoting cell proliferation, accelerating angiogenesis, reducing infection, and increasing collagen synthesis [204, 205]. The oxygenation level in the wound microenvironment is a key rate-limiting factor for wound healing [206, 207]. Long-term hypoxic conditions lead to impaired neovascularization and limited wound healing [208], whereas a hypoxic environment promotes wound healing [209]. Therefore, in situ oxygen production, oxygen carriers, and various strategies to enhance oxygen supply have been used to relieve the hypoxic state of wounds, promote angiogenesis, and enhance collagen remodeling, ultimately accelerating wound healing [210–213].

### Reducing oxidative stress

Diabetes mellitus (DM) is a chronic disease that affects millions of humans. Further, in more than 25% of patients with DM, factors such as uncontrollable bacterial infections [214], persistent inflammation [215], and hypoxia [216] can cause foot ulcers, which, in severe cases, can lead to amputation [217, 218]. According to previous studies, the long-term exposure of diabetic patients to a high glucose environment can functionally impair the of HIF-1α/ VEGF axis, resulting in reduced abilities to reverse and regulate the ischemic hypoxic state of the wound tissue [207, 219] to promote wound healing. Fukui [220] et al. suggested that LEH has the advantages of small particle size and high oxygen affinity, effectively perfusing obstructed blood vessels and improving the oxygen supply to the wound. Studies based on a diabetic mouse (dB/ dB) skin defect model have shown that h-LEH significantly increased the oxygen supply level of the wound microenvironment and inhibited the inflammatory cascade [221] while effectively improving the microcirculation and accelerating diabetic wound healing. MXene is a two-dimensional material with ultrathin layer topology and good biocompatibility [222]. Li et al. [79] used a system of H_2_O_2_/HbO_2_ to catalyze the oxidative coupling of dopamine-grafted hyaluronic acid and PDA with encapsulated Ti_3_C_2_MXene nanosheets to form injectable hydrogels (Ti_3_C_2_@PDA or MXene@PDA NSs). HbO_2_ exert HRP-like enzyme activity for catalyzing gel formation, whereas MXene scavenge excess free radicals, reduce oxidative damage, and maintain oxidative homeostasis while exerting a bacteriostatic effect [223]. Further, MXene and PDA have excellent photothermal conversion features that can generate heat to elevate temperature under 808 nm NIR irradiation, thereby achieving a Hb-reversible oxygen release in the wound microenvironment. In the dB/dB wound model, the hydrogel oxygen carrier continuously relieved the hypoxic state of the wound tissue, reduced oxidative stress, regulated macrophage polarization, promoted angiogenesis, and accelerated the healing of diabetic wounds.

### Promoting tissue repair

**Fig. 7:**
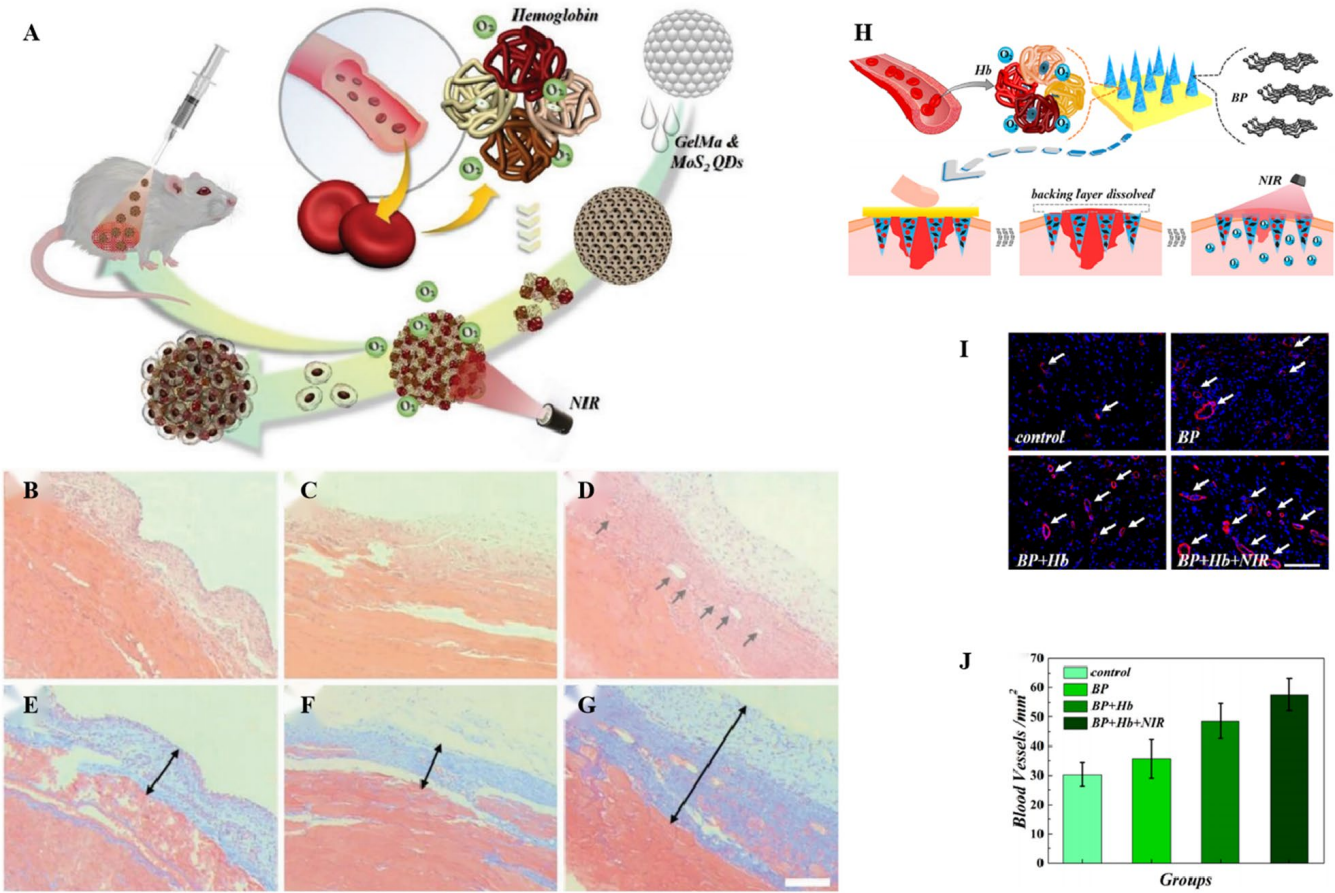
**A** The schematic illustration of the light-responsive MoS2 QDs integrated inverse opal microcarriers for controllable oxygen delivery and tissue repair. The H&E staining of **B–D** repaired samples after implantation for 2 weeks. Vessels in the samples are indicated with grey arrowheads. **B** Control, **C** Experimental I, **D** Experimental II. The Masson staining of **E–G** repaired samples after implantation for 2 weeks. Granulation tissue thickness in the samples are indicated with black arrowheads. **E** Control, **F** Experimental I, **G** Experimental II. **B–G** The scale bar is 200 µm. **A–G** Reproduced with permission [233]. [88]. Copyright 2020, American Chemical Society Nanomaterials. Copyright 2019, small. **H** Schematic illustrations of wound healing using NIR responsive separable MNs which encapsulate BP QDs and oxygen-carrying Hb. **I** Corresponding double immunofluorescent staining of CD31 and α-SMA on day 9. The arrows indicate the vascular ducts. The scale bars are 100 μm. **J** Corresponding quantitative analysis of the blood vessel density on day 9. The scale bars are 50 μm. **H–J** Reproduced with permission

O_2_ promotes tissue formation and repair, thereby accelerating wound healing [224, 225]. Currently, the development of miniature, universal, and customizable O_2_ carries is progressing rapidly [226, 227] and holds great potential for applications in the field of wound healing. Liu et al. [228] combined molybdenum disulfide quantum dot-blended gelatin methacryloyl (GelMa) to produce inverse opal microparticles as microcarriers for preparing NIR-triggered porous controllable oxygen carriers by coupling amide bonds with bHb for tissue repair. Based on a typical abdominal wall defect rat model, this oxygen carrier was found to improve the oxygen supply and play a role in supporting cell growth, thereby stimulating extracellular matrix secretion, promoting collagen and angiogenesis, accelerating granulation tissue formation, and promoting the repair of abdominal wall defects. The characteristics of oxygen carriers in tissue repair render them useful for wound healing and tissue engineering (Fig. 7).

Zhang et al. [86] constructed responsive microneedles (MNs) simultaneously loaded with black phosphorus (BP) and hHb to overcome the limitation that most oxygen carriers can only contact the surface area of the wound without reaching the inner tissue. These responsive MNs could penetrate the epidermal layer to the interior of wound tissue in a painless, noninvasive, and sterile manner [229, 230], thereby achieving controllable delivery of O_2_ and promoting wound healing. Further, a NIR controllable O_2_ releasing carrier was fabricated using GelMA as a synthetic material for responsive MNS, which helped improve the mechanical strength and penetration ability of MNs loaded with quantum dots of BP and Hb. In a full-thickness cutaneous wound type I diabetes rat model, the treatment group exhibited the smallest wound area, narrowest wound bed, thickest regenerated epithelial tissue, and densest neovascular state, indicating excellent healing capacity compared to the control groups. These results suggest that this responsive MN oxygen carrier proposed by this research group has an ideal therapeutic effect on wound healing.

Currently, there are only a limited number of studies on the application of Nano-HBOCs to wound healing, and the main forms of drug delivery primarily involve hydrogels and microneedles. By expanding the options for administration, such as through spraying, we believe that Nano-HBOCs will be able to better meet the clinical requirements of wound healing and become an effective therapeutic method.

## Nano-HBOCs for other biomedical applications

Nano-HBOCs have also been used in other disease therapies, as discussed below.

Port-wine staining (PWS) is a non-neoplastic malformation caused by telangiectasia at birth [231] that mostly occurs in the head and neck region and severely affects patient penetrance and self-confidence [232, 233]. PWS grows modularly, tends to bleed [234], and forms fibrous scars on the skin surface, which become more pronounced after puberty [235]. Currently, PDT is a primary treatment strategy for PWS [236]. HbVs are smaller (250 nm) than RBCs and 1/40 the diameter of RBCs; further, they are inclined to flow in the marginal zone of microvessels in the blood [237] and increase the hemoglobin concentration in microvessels, enabling a more efficient conversion of light energy into heat energy. Rikihisa et al. [238] applied a laser as a treatment for PS in PWS (at a wavelength of 595 nm) using the oxygen-carrying capacity of HbVs (or LEHs). An oxygen-rich environment enhanced the PDT effect, generated a large amount of ROS, damaged the vessel wall, treated PWS, and achieved a better therapeutic effect in an animal model with chicken wattle as PWS.

Pre-eclampsia (PE) can lead to pre-term birth in pregnant women and is a leading cause of maternal and fetal death [239–241]. Studies have shown that inadequate trophoblast invasion in the placenta leads to the narrowing of the spiral arteries of the placenta, thereby causing placental ischemia/ hypoxia and limiting gas and nutrient exchange between the mother and fetus, which ultimately causes intrauterine growth restriction, maternal hypertension, and organ damage [242–246]. HbV, which is a type of Nano-HBOC with a nanoscale size and no blood group antigens, can pass through narrow placental spiral arteries and capillaries to provide an efficient O_2_ supply to the placenta [247, 248]. Ohta et al. [249] intravenously injected HbV into a rat preeclampsia model. The results showed that HbV alleviated fetal hypoxia by supplying oxygen while improving fetal growth restriction in a murine model of preeclamptic pregnancy. This study suggests that HbV is an effective treatment modality for fetal hypoxia caused by placental dysfunction during pregnancy.

*Porphyromonas gingivalis*, a gram-negative anaerobic bacterium, is one of the main pathogens causing oral infections (including gingivitis, periodontitis, and oral ulcers) [250]. Traditional broad-spectrum antibiotic therapies often lack selectivity and can lead to oral flora dysbiosis [251]. Previous studies have shown that *P. gingivalis* can bind to and acquire porphyrin molecules from Hb; therefore, Hb can be used as a specific drug delivery vehicle to treat *P.gingivalis* infection [252]. Based on this idea, Bai et al. [253] complexed rat oxyhemoglobin (oxyHb) with an anionic amphiphilic PS (IR820) to produce a stable nano-photosensitizer oxyHb@IR820. oxyHb@IR820 targets *P.gingivalis*, mediating the synergistic antibacterial effects of photothermal therapy and PDT [254–256] and providing a new therapeutic strategy against a *P.gingivalis* infection. In an oral infection model of *P.gingivalis* in rats, oxyHb@IR820 supplied exogenous O_2_ and addressed the limitation caused by the poor effectiveness of PDT on anaerobic bacterial infection in hypoxic environments. Meanwhile, the specific uptake of P*.gingivalis* oxyHb@IR820, which improved the photodynamic and photothermal conversion efficiencies, significantly inhibited bacterial growth and reduced the oral ulcer area.

Further, Nano-HBOCs showed great application prospects in other tissue engineering scenarios. For example, Paciello et al. [257] developed a novel smart cyclic oxygen-releasing biomaterial called G-HbOD, which demonstrated superior biocompatibility and oxygen supply capability for maintaining cell viability under hypoxia in 3D cell culture tests.

Vasosuppression [258] and oxidative stress [259] resulting from hypoxia are major obstacles to bioartificial islet (BAP) transplantation [260]. To address this issue in human islet allotransplantation, Mouré et al. [261], encapsulated marine worms (HEMOXCell) Hb [262], via alginate and silicone calcium peroxide, creating a compound that produced, carried, and released O_2_, increasing the O_2_ concentration in BPA. This strategy effectively addressed the lack of O_2_ within the graft in a closed environment, and promoting neovascularization. This novel technology provides innovative solutions for tissue and bioengineering O_2_ supply. Enzyme biofuel cells (EBFC) are widely used as implanted biomedical devices in cardiac pacemakers [263], electrical sensors [264], and biosensors for physiological parameters [265]. However, the current catalysts for EBFC have very low catalytic performance and poor adhesion on the electrode surface. Chen et al. [266] anchored Hb on exogenous Ca^2+^ and PO3-4 to generate hydroxyapatite (Ca_10_(PO_4_)_6_(OH)_2_, HAP) nanodots and reported a novel EBFC constructed from nanoengineered RBCs (NERBCs). Given its strong oxygen adsorption capacity, NERBCs exhibits excellent catalytic reduction capacity, biocompatibility, high selectivity, and lifespan when used as an EBFC-negative catalyst, thereby pointing to a new direction for fabricating bio-nanobatteries.

## Conclusions and perspectives

O_2_ plays a vital role in life activities, and Hb is a natural carrier of oxygen in the body. The timely transfusion of RBCs can effectively restore blood volume and maintain tissue oxygen supply in both clinical and military treatment scenarios. HBOCs have become the main focus for artificial oxygen carriers due to their advantages of being closer to natural Hb and providing an effective method to solve the inherent problems of natural blood transfusion. The rapid development of nanotechnology and nanomaterials has opened new possibilities for HBOCs.

Nano-HBOCs are a new type of hemoglobin-based oxygen carriers developed using nanotechnology combined with nanomaterials. This combination, has helped improve the application scenarios of Hb and has shown certain advantages in enhancing the stability of the molecular structure of Hb, reducing vasoactivity, prolonging half-life, and improving biocompatibility. Furthermore, Nano-HBOCs have broad application prospects (Table 2), and have attracted increasing attention from the medical, material, chemical, biological, and engineering research attention. Despite the rapid development of Nano-HBOCs in the field of biomedical research, there are still several limitations associated Nano-HBOCs, which are listed below: Nano-HBOCs require complicated preparation processes and are associated with prohibitive costs. The development of simple, safe, and cost-effective preparation methods will be the focus of future studies.

1. Constructing Nano-HBOCs with characteristics and functions similar to those of natural RBCs is difficult; more in-depth systematic research and technological progress are required to achieve a complete biomimetic replacement of natural RBCs.
2. Clinical transformation and applications are significantly challenging. Currently, the vast majority of Nano-HBOCs are in the laboratory research stage, and relevant clinical research is yet to be conducted. Therefore, it is necessary to conduct strict and systematic safety and perform effective evaluations for diverse application scenarios to meet the regulatory requirements.
3. Non-functional methemoglobin poses a significant challenge in the development of Nano-HBOCs. However, existing studies have not sufficiently addressed the control of Hb oxidation and lacked molecular characterization. In our review, we emphasize the need to pay attention to methemoglobin production. Furthermore, we propose various approaches to address this issue, including low-temperature operation, reducing the reaction time, adding antioxidants, and employing carbon-bonding techniques etc. in the further, we hope to develop simpler and more efficient methods for controlling and detecting methemoglobin to explore more simple and efficient methods for the control and detection of methemoglobin.
4. Biosafety is crucial in development of Nano-HBOCs for use in the treatment of various diseases. This is particularly important in scenarios involving high doses and multiple infusions, where in vivo metabolism, transportation, toxicity evaluation, and clearance of by-products of Nano-HBOCs are major concerns. However, there is a lack of standardized evaluation methods for hemoglobin biocompatibility, especially in tests such as antigenicity and complementation experiments. Therefore, a comprehensive and systematic standardized evaluation of Nano-HBOCs biocompatibility is essential for complete replacement of natural erythrocytes.

**Table 2:** The key points of Nano-HBOCs, including approaches to fabrication, targeted fields and references

Key Points	Categories	Approaches	Targeted Fields	References
Liposome-encapsulated	TRM-645	PEG modify liposomes which mimicking the RBCs membrane	Hemorrhagic shock	[69]
	Erythromer (EM)	A novel shuttle, which adjust P_50_ in response to pH changes		[107]
	HbPs	Hb encapsulated mPEG-PLGA through double emulsion technique		[70]
	LHE/HbV	Hb encapsulated by different type of vesicles	Hemorrhagic shock Ischemic stroke Cancer Wound healing PWS PE	[104, 137, 166, 220, 249, 267]
PDA surface-coated	Hb-PDA	Using a template-based co-precipitation technique	Hemorrhagic shock	[77]
	PDA − LtEc	A photocatalytic method		[73]
	CPTK@PMH	Engaging Hb and M to form PMH, connecting to a specific fibrin-binding peptide	Ischemic stroke	[81]
	MXene@PDA	Hyaluronic acid and PDA catalyzed by H_2_O_2_/HbO_2_ to encapsulated Ti_3_C_2_MXene	Wound healing	[79]
ZIF-8	ZIF-8@Hb	MOFs	Ischemic stroke	[74]
	Hb@AuNCs	Synthesizing ultrasmall protein metal clusters	Cancer	[75]
	O_2_@Hb@ZIF-8	MOFs		[72]
Core–shell structural	HemoAct	Conjugating one Hb molecule with three HSA	Ischemic stroke	[140] [142]
	Hb-HSA_3_(PtNP)	Combining platinum nanoparticles with HSA		
	C@HPOC	Protein hybridization	Cancer	
				[193]
Others	H-NPs	Combining Hb with DAC	RCC	[169]
	V(Hb)@DOX	Coupling PCL self assembles to form hollow Nanovesicles	Cancer	[84]
	HCM	PMAG linking GLUT1		[82]
	DHCNPNPs	Hb and DOX encapsulated in PLGA		[83]
	Au-Hb@PLT	Synthesized by coating Au-Hb NPs with PLT membrane		[85]
	P-FRT-RBC	Ferritin, a protein-based nanocage		[76]
	BP@RB-Hb	Assembling Hb into nanocomplexes with BP and PS		[192]
	OxyHb@IR820	oxyHb complexed with an anionic amphiphilic PS	P. gingivalis	[253]
	MN	Loading with black phosphorus	Wound healing	[86]
	G-HbOD	Conjugating human Hb to the surface of gelatin microspheres	3D cell culture	[257]
	NERBC	Anchoring Hb on exogenous Ca^2+^ and PO_4_^3-^	Bio-nanobatteries	[266]

We firmly believe that further research on Nano-HBOCs holds immense potential for novel advancements and significant breakthroughs in multiple fields. This promising area of study not only exhibits great prospects for practical applications but also demonstrates the potential to revolutionize clinical practical and significantly enhance human health.

## Data Availability

Not applicable.

## Abbreviations

AA: Arachidonic acid
BAP: Bioartificial islet transplantation
BP: Black phosphorus
bHb: Bovine hemoglobin
DHCNPNPs: Camouflaged biomimetic nanocomposites
CAT: Catalase
Ec: Cell-free hemoglobin
DAC: Decitabine
DHL: DOX-Hb-lipo
DM: Diabetes mellitus
EBFC: Enzyme biofuel cells
EM: Erythromer
FDA: Food and drug administration
G-HbOD: Gelatin hemoglobin depot
GelMa: Gelatin methacryloyl
GM: Gelatin microspheres
GLUT1: Glucose transporter isoform 1
HCM: Hb-conjugated micelles
Hb: Hemoglobin
HbV: Hemoglobin vesicles
HBOCs: Hemoglobin-based oxygen carriers
HbPs: Hemoglobin-loaded nanoparticles
HbTcMs: Hemoglobin-polymer conjugate
HS: Hemorrhagic shock
HDAS: Hexadecyl carbamoyl methyl hexadecanoate
V(Hb): Hollow nanovesicles
HeLa: Human cervical cancer cells
hHb: Human hemoglobin
HSA: Human serum albumin
HIF-1α: Hypoxia inducible factor-1α
HRP: Horseradish peroxidase
ICG: Indocyanine green
LEH: Liposome-encapsulated human hemoglobin
h-LEH: Liposomes encapsulating hemoglobin
RCC: Renal cell carcinoma
Lt: *Lumbricus terrestris*
MMP-9: Matrix metalloproteinase-9
MPA: Mean arterial pressure
MOFs: Metal–organic frameworks
MNs: Microneedles
MCAO: Middle cerebral artery occlusion
MnPcs: Mn-phthalocyanine
CT-26: Mouse colon cancer models
MDR1: Multidrug resistance gene 1
NERBCS: Nanoengineered red blood cells
NEMR: Nanoerythrocytes
Nano-HBOCs: Nanomaterial-related HBOCs
I-ARCs: Nanoscale artificial blood cells
NIR: Near-infrared
OHZ: O_2_@Hb@ZIF-8
OXA: Oxaliplatin
oxyHb: Oxyhemoglobin
PFCs: Perfluorocarbons
P-gp: P-glycoprotein
PDT: Photodynamic therapy
PDT: Photodynamic therapy
PS: Photosensitizer
PLT: Platelets
PDA: Polydopamine
PWS: Port-wine staining
PE: Pre-eclampsia
RT: Radiation therapy
ROS: Reactive oxygen species
RBCs: Red blood cells
FRET: Resonance energy transfer effect
SDT: Sonodynamic therapy
SOD: Superoxide dismutase
tMCAO: Transient MCAO
TME: Tumor microenvironment
TAM: Tumor-associated macrophage
ZnPc: Zinc phthalocyanine
